# StrataSeq: A Workflow for Rapid Development of Molecular Databases for Hard‐To‐Identify Species

**DOI:** 10.1002/ece3.72375

**Published:** 2025-10-23

**Authors:** Anna K. Merges, Peter Manning, Dennis Baulechner, Katharina John, Andrey Zaitsev, Volkmar Wolters, Damian Baranski, Hans‐Peter Grossart, Jason Woodhouse, Clément Schneider, Miklós Bálint

**Affiliations:** ^1^ Institute of Insect Biotechnology Justus‐Liebig University Giessen Germany; ^2^ Senckenberg Biodiversity and Climate Research Centre Frankfurt am Main Germany; ^3^ LOEWE Centre for Translational Biodiversity Genomics Frankfurt am Main Germany; ^4^ Department of Biological Sciences University of Bergen Bergen Norway; ^5^ Institute of Animal Ecology Justus‐Liebig‐University Giessen Germany; ^6^ Senckenberg Museum of Natural History Görlitz Görlitz Germany; ^7^ Department of Plankton and Microbial Ecology Leibniz Institute of Freshwater Ecology and Inland Fisheries (IGB) Stechlin Germany; ^8^ Institute of Biology and Biochemistry Potsdam University Potsdam Germany; ^9^ Institute of Cell‐ and Systems Biology of Animals University of Hamburg Hamburg Germany

**Keywords:** biodiversity monitoring, DNA reference database, habitat‐stratified sampling, morphological identification, morphospecies, species turnover

## Abstract

Biodiversity loss necessitates improved monitoring of small, species‐rich taxa, such as protists, phyto‐ and zooplankton and terrestrial invertebrates. Traditional biomonitoring is often infeasible for these taxa due to complex morphology and few taxonomists. DNA‐based approaches offer promising solutions by enabling rapid species identification. However, the effectiveness of these methods depends on the completeness of molecular reference databases, which remain incomplete, particularly for remote and biodiverse regions. To address this, we propose the StrataSeq workflow, a systematic approach to optimise the generation of DNA reference databases for hard‐to‐identify taxa. Reference sequences allow us to connect molecular operational taxonomic units to a wealth of information available for many described taxa. StrataSeq consists of four key steps: (1) Habitat‐stratified sample subsetting selects a minimal but ecologically representative sample set by stratifying along key environmental gradients. (2) Prioritising morphospecies involves sorting specimens into morphospecies and ranking them based on their occurrence across samples, prioritising common taxa for detailed identification. (3) Detailed morphological identification focuses on common morphospecies to maximise taxonomic coverage while minimising effort. (4) Reference DNA sequence generation targets taxa lacking molecular references, with sequenced specimens deposited as museum vouchers. We benchmarked the StrataSeq workflow using two datasets of Collembola from grassland soils in Germany. In comparison with a species list generated by a more labour‐intensive traditional approach (identification of randomly selected individuals from all samples), the StrataSeq workflow captured 69% of species but required only 22% of the effort. StrataSeq is adaptable to various organism groups and environmental settings, including both spatial and temporal gradients. The workflow enhances the cost‐effectiveness of generating reference DNA databases, supporting improved biodiversity monitoring and ecological research. StrataSeq offers a scalable solution to accelerate the completion of molecular databases, thereby improving biomonitoring and ecosystem assessments under global change pressures.

## Graphical abstract

Biodiversity loss calls for better monitoring of small, species‐rich taxa, such as soil invertebrates, but traditional methods are limited due to complex morphology and lack of expertise. DNA‐based approaches offer a solution, but their effectiveness depends on incomplete molecular reference databases. The StrataSeq workflow optimises DNA reference generation through habitat stratification, prioritising common species for detailed identification and sequencing specimens lacking molecular references, making biodiversity monitoring more efficient and cost‐effective, as shown by its successful application to Collembola in German grasslands.

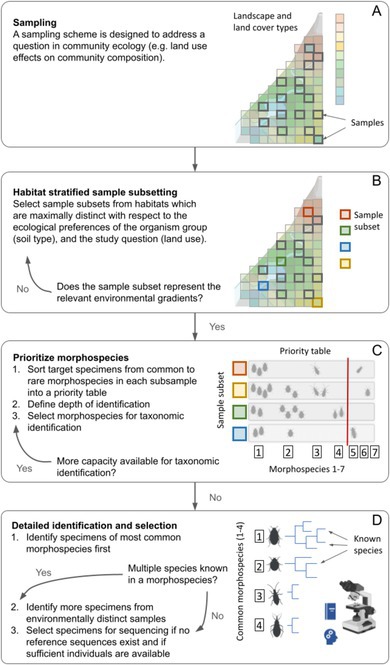

## 1 Introduction

Accelerating biodiversity loss necessitates greatly improved monitoring of biodiversity trends across spatial and temporal scales (Gonzalez et al. 2023). Small and species‐rich taxa, such as protists, phyto‐ and zooplankton, zoobenthos, small terrestrial invertebrates, but also bacteria and fungi, constitute most of global biodiversity (Wiens 2021). However, adequate biomonitoring of small taxa using traditional approaches is often infeasible as their detection and morphological identification are difficult and time‐consuming (Oliver and Beattie 1993; Terlizzi et al. 2003), and taxonomic expertise is increasingly rare (Hutchings 2019). Recently developed DNA‐based approaches may become cost‐effective tools for rapid and widespread assessment of hard‐to‐identify taxa (Elbrecht et al. 2017; Porter and Hajibabaei 2018). These methods allow us to identify species and characterise community compositions utilising DNA sequences specific to species. However, the molecular identification of species relies on the availability of taxonomically annotated DNA sequence databases. These databases are necessary to link DNA sequences to described species and to knowledge of their functions, interactions, or sensitivity to stressors.

Many initiatives have contributed to the creation and expansion of molecular reference databases. Such initiatives include the Barcode of Life Data System (BOLD) (Ratnasingham and Hebert 2007), the Earth Biogenome Project (Lewin et al. 2022), SILVA (Quast et al. 2013), which is specialised on ribosomal DNA sequences, and Unite for fungi (Abarenkov et al. 2010). Despite these advances, existing databases are far from complete. Data gaps for both genome and morphological data increase with increasing geographical remoteness (Monchamp et al. 2023), including some of the most biodiverse regions, like the tropics (Arana et al. 2024). Data incompleteness is a major problem for hard‐to‐identify taxa where a lack of taxonomic expertise limits morphological identification. This means reference databases contain only limited numbers of sequences for most hard‐to‐identify taxa. For instance, Li et al. (2022) found that 70% of aquatic insects from Chinese rivers miss molecular references. Gaps of terrestrial arthropods were reported by Recuero et al. (2024), who found that taxonomically informative DNA sequences are available only for a few Appalachian soil arthropods. Sequence data deficiency limits our capacity to currently use DNA‐based tools to monitor and understand the ecology of the most hard‐to‐identify taxa.

Incomplete sequence databases mean that research projects targeting hard‐to‐identify taxa, or groups in geographically remote regions, are confronted with a choice: they either base their conclusions on taxonomy‐free approaches (Cordier et al. 2019), or they have to generate new reference libraries themselves (Lopes‐Lima et al. 2024; Shechonge et al. 2024). If researchers choose database generation, covering all target taxa in the region of interest is likely impossible. Consequently, it is necessary to prioritise certain aspects of database creation to limit the most laborious tasks: sample sorting and identification (often through microscopy), while creating new reference data that is still representative of the investigated species and communities. Prioritisation strategies may promote pragmatic workflows that balance taxonomic ambition with feasibility in biodiversity assessments (Meier et al. 2025).

Numerous ecological studies have already dealt with the question of how to optimise species sampling for improving time‐ and cost‐efficiency (Bennett et al. 2014; Meyer et al. 2021; Milián‐García et al. 2021). When aiming to compile a species inventory of a region while minimising the number of samples to sort through, one frequently applied method to optimise species detection is a stratified sampling approach (Danz et al. 2005). In this approach, a study area is divided into layers (strata) based on certain environmental characteristics, such as habitat types, from which samples are subsequently picked for adequate representation of subgroups. This idea is based on the ecological assumption that habitat heterogeneity generates turnover in species composition: species have unique niches, and consequently, species composition should strongly differ along major environmental gradients (Hutchinson 1957). Sample selection strategies based on habitat heterogeneity, therefore, promise to maximise the number of detected species. Ideally, direct measurements of important parameters, such as water or soil chemistry, should be used to account for small‐scale spatial heterogeneity. Open access environmental data (like climate variables, soil types, vegetation types, elevation) (Palmer et al. 2002). However, stratified sampling strategies have not yet been applied to sequence database creation.

The ability to deal with high specimen abundances is another important optimization aspect. As samples of small organisms often contain thousands of target specimens, sorting them by morphospecies is a common method to gain a rough estimate of taxonomic diversity. Morphospecies sorting is the classification of organisms into at least superficially similar groups on the basis of external morphology, without the consultation of taxonomic literature or experts (Krell 2004). Morphospecies are not sufficiently informative about taxonomic identity. However, the sorting is often a useful first step prior to accurate downstream species identification as morphospecies can help streamline species identification efforts. Importantly, the sorting allows one to identify common morphospecies, i.e., those that constitute most of the target community. Here we define ‘common’ as taxa widely occurring across different habitat types. A collateral macroecological tendency of widely occurring taxa is that these often occur in high abundances (Verberk 2011). Common taxa usually contribute more to the functioning of their ecosystem (Grime 1998; Smith and Knapp 2003), although rare species can also be functionally important (Litchman et al. 2024; Mouillot et al. 2013). Prioritising common morphospecies for downstream morphological identification has the advantage that identification effort can be weighed against cost‐ and time‐efficiency. Such ‘stop‐rules’ are crucial to consider when dealing with limited resources (Cardoso 2009). Identification efforts might also be reduced by identifying taxa only to higher levels (e.g., to genera or families, instead of species). However, information loss from relaxed taxonomic resolution is potentially greater compared to information loss from a reduced number of identified specimens (Bennett et al. 2014).

While concepts for optimising sampling efficiency are frequently used in community ecology and taxonomy, they have not been adapted into a conceptual workflow for optimising the generation of DNA sequence databases. There is a need for such frameworks as more and more ecological studies and biomonitoring efforts rely on molecular morphological identification. Here we propose the StrataSeq workflow to optimise the generation of new molecular references for hard‐to‐identify and species‐rich taxonomic groups. The workflow relies on (1) stratified sampling across environmental driver gradients to optimise the recovery of taxonomic diversity, (2) prioritising common over rare taxa to generate reference sequences for most individuals of targeted communities, (3) existing taxonomic knowledge when prioritising morphospecies for detailed morphological identification, and (4) proposing a species list for reference sequence generation. We benchmark the StrataSeq workflow against the traditional method with a Collembola dataset where all specimens are identified prior to sequencing selection.

## 2 Materials and Methods

### 2.1 Description of Workflow

StrataSeq is primarily a conceptual workflow that involves human judgement at critical decision points rather than automated computational steps. Therefore, computational scripts are limited to visualisation and ordination analyses and common ecological procedures.

#### 2.1.1 Habitat Stratified Subsetting

**FIGURE 1:**
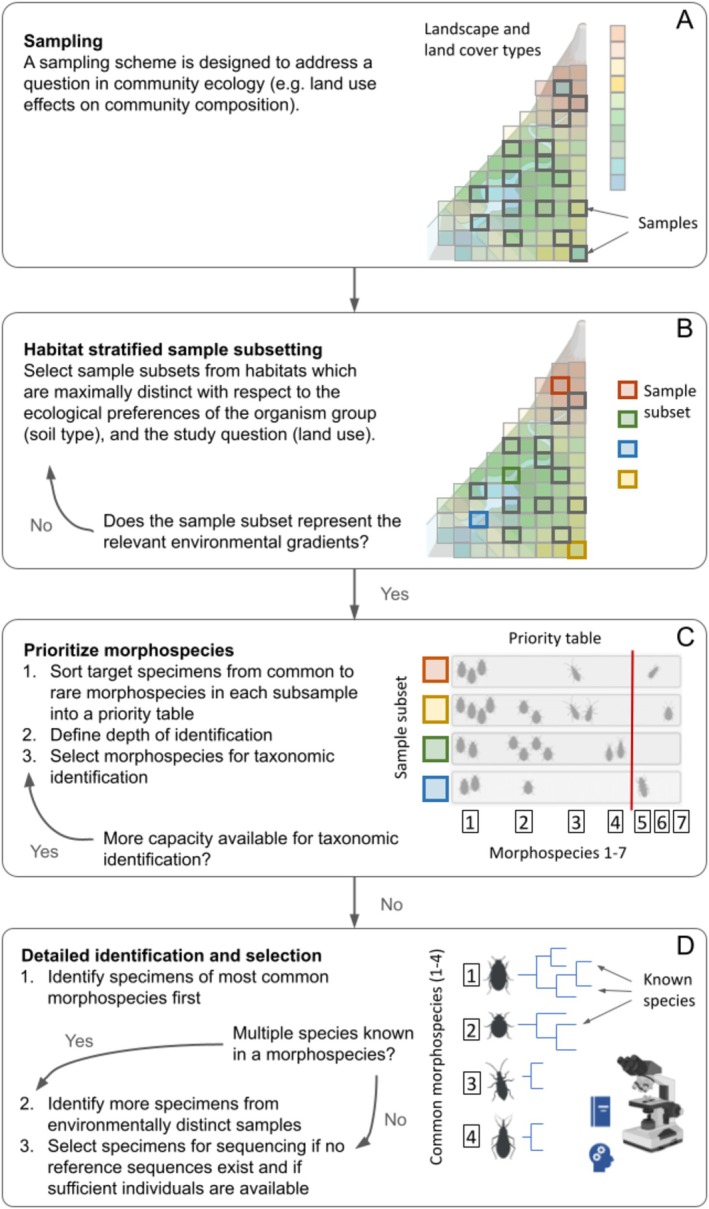
Overview of the workflow. (A) Sampling a community to answer an ecological question. Thick rectangles represent all samples taken. (B) Habitat‐stratified sample subsetting: A subset of samples is chosen based on environmental variables (strata) that are expected to drive compositional turnover in the target communities (= habitat‐stratified sample subset). Coloured rectangles represent samples designated for reference sequence generation after the habitat‐stratified sample subsetting. (C) Prioritising morphospecies: From the sample subset all specimens are sorted into pools of morphospecies. These pools are kept separated per sample. Individual IDs (i.e., unique codes given to each morphospecies, here as framed numbers), numbers of specimens per morphospecies, numbers of morphospecies pools per sample are noted in a morphospecies‐sample matrix. Morphospecies are sorted from common to rare. A stop‐rule is defined for detailed identification according to available capacities (red line). Here, four morphospecies are selected for detailed identification: 1 (common), 2 (common), 3 (common), and 4 (not common, but abundant in a sample). (D) Detailed morphological identification: Specimens of common morphospecies are identified first, followed by increasingly rare morphospecies. If a morphospecies may contain multiple species, specimens are identified from multiple samples which represent maximally different habitats. Reference DNA sequences are generated for species without publicly available reference data.

As a starting position, we assume we have a full set of samples for molecular identification, obtained from a sampling scheme designed to answer an ecological question about community composition. To minimise effort in sorting and identifying species, we subset the samples by stratifying them along environmental gradients known or expected to be important in driving species turnover of the target taxa (e.g., geographic region or soil type; Figure 1). The number of samples to be sorted is kept at a minimum, but the maximised environmental heterogeneity among the samples increases the chances of finding a large fraction of the entire species pool. With this strategy, we select samples across the broadest possible range of differing environmental gradients, assuming that biodiversity and species composition change most rapidly across major habitat differences. At the end, we intend to have a single representative sample for each stratum. If we end up with multiple samples per stratum, we can take another level of stratification or randomly choose one from the samples. We term the set of samples resulting from this step as the habitat‐stratified subset. While we here describe the approach for spatial sampling schemes, it can be adapted to temporal assessments, e.g., seasonal community changes. If sampling was done over multiple time points, the samples may be subsetted also by time points representing the most important temporal environmental changes (e.g., temperature, vegetation period, hours of light).

#### 2.1.2 Prioritising Morphospecies

The second step is to sort all specimens within the habitat‐stratified sample subsets into morphospecies. These morphospecies are then ranked by their commonness across the samples, i.e., the number of samples they are present in. The more common a morphospecies, the higher its priority is for detailed morphological identification (Figure 1 and Table S1) (Merges et al. 2025). While we prioritise morphospecies which appear in the largest number of samples, a distribution‐abundance relationship predicts that widely distributed species tend to be also locally abundant (Verberk 2011). The number of specimens of each morphospecies should be noted within a sample, along with the number of intact and adult specimens. This will help select morphospecies pools with sufficient individuals for detailed morphological identification and sequencing (see below). When several morphospecies are equally common, abundance of specimens can be taken as a second prioritisation argument, i.e., the morphospecies with the higher number of specimens is prioritised. This is especially relevant when the identification effort has progressed towards rarer morphospecies and the work capacity for identification is close to the limit. Morphospecies of remarkably high abundance within a single or very few habitat subset samples should be marked and prioritised when workload capacities allow identification of rarer taxa. The task of sorting the morphospecies can be done by non‐experts. In such cases, training on possible morphological variance of a target taxon (e.g., known life cycle and phenotypic variation in size, shape and colour) should be given in advance by an expert. We also recommend taking high resolution pictures of the specimens in the bulk samples: this will allow us to get a second opinion on ambiguous cases later and may facilitate cross‐sample morphospecies comparison. The outcome of this step is a set of morphospecies pools from each habitat‐stratified sample, along with a corresponding abundance matrix (morphospecies‐sample matrix).

#### 2.1.3 Detailed Morphological Identification

This step is the most labour‐intensive, and consequently it is the main bottleneck for reference sequence database creation. Taxonomic literature (e.g., identification keys) and taxonomic expertise are essential here. Specimens should be prepared and identified according to the morphospecies priority table (Figure 1): the number of adult and intact specimens per pool of morphospecies, considering also habitat differences between the stratified samples. If a morphospecies is common in many samples, specimens should be first identified from the morphospecies pools of the most distinct habitats to minimise redundancy in identified species. At the same time, it is important to consider the number of specimens needed for confident species identification and DNA sequence generation. For many organism groups, reliable identification and sequencing both may need more than one specimen: multiple microscopic slides might be necessary with specimens mounted at different angles, and multiple DNA extractions might be needed for sufficiently high‐quality DNA. Consequently, the number of available specimens is especially important for morphospecies that are known to be species‐rich. In general, the more individuals are available in a pool of a morphospecies, the higher the chances are for clear species identification and good‐quality DNA extracts. The number of specimens necessary for robust identification and DNA extraction will largely depend on body size, preparation skill, or the type of distinguishing morphological characteristics. There might be cases when morphological identification and DNA extraction are not possible on the same specimens. In this situation, specimens for DNA extraction should be selected from a morphospecies pool where a single taxonomic species dominated in a habitat‐stratified sample.

#### 2.1.4 Reference DNA Sequence Generation

Once the identification step is finished, the list of species should be checked against public molecular reference databases. Specimens should be designated for sequencing, i.e., those identified species that lack molecular references of the desired type or quality, e.g., barcodes or genomes (Figure 1). For species belonging to multi‐taxon morphospecies (i.e., those that may contain multiple species), either parts of the body of a well‐identified specimen should be used for DNA extraction (if organisms are sufficiently large), or the body should be recovered after DNA lysis (Gilbert et al. 2007) and microscopically checked to make sure that the taxonomic assignment of the sequenced specimen is correct. In the case of genome sequencing, we recommend that a single specimen is used because hidden genetic variation may render genome assembly difficult if multiple genetically distinct specimens are pooled. Specimens with body parts sequenced, or bodies recovered after DNA extraction should be deposited as vouchers in publicly accessible specimen collections. This is essential for future verification (Buckner et al. 2021).

### 2.2 Benchmarking of StrataSeq

In the following section we demonstrate the workflow using two datasets of Collembola obtained from soil samples taken from grassland plots in Germany (StrataSeq dataset). These 150 grassland plots are part of the Biodiversity Exploratories, a long‐term observational and experimental network set up to investigate the impacts of land‐use intensification on biodiversity (Table S2) (Fischer et al. 2010). We compare specimens identified with the StrataSeq workflow with a second, benchmarking dataset generated by identifying a random set of specimens in all samples. The benchmarking dataset was generated by morphologically identifying specimens from all plots from the same grasslands. Field permits for the collection were issued by the responsible state environmental offices of Baden‐Württemberg, Thüringen and Brandenburg.

Collembola are a large group of globally occurring terrestrial microarthropods (1–5 mm length on average). Approximately 9000 species have been described to date. Their communities often reach abundances of hundreds of thousands of individuals within a square metre of soil (Coleman et al. 2020). Collembola feed on a wide variety of different resources, such as leaf litter, fungi, bacteria, roots and algae. Consequently, they are important regulators of microbial biomass and influence soil nutrient cycling as well as soil structure (Coulibaly et al. 2019; Potapov et al. 2020; Rusek 1998).

The samples for both datasets were taken from the 150 grassland plots of the DFG Biodiversity Exploratories long‐term priority program (Table S2) (Ostrowski et al. 2024). The 150 plots are spread across three regions in Germany, with 50 plots located each in the Swabian Albs, Hainich‐Dün and Schorfheide‐Chorin. The plots are selected to span the full grassland land‐use intensity (LUI) gradient of each region (Table S2) (Fischer et al. 2010). LUI is calculated as the sum of the standardised values for three primary management practices: mowing events per year, amount of fertiliser applied, and livestock units per hectare per year (Blüthgen et al. 2012). According to Wolters et al. (2023), on average, 4.5 species are present on the Swabian Albs plot (0–9 species), 2.5 species on the Hainich plots (0–7 species) and 2.6 on the Schorfheide plot (0–7 species).

### 2.3 Benchmarking Dataset

The sampling for benchmarking taxa was done in April 2019 with the aim of characterising the collembola communities of all 150 plots of the Biodiversity Exploratories. Nine soil cores (5 cm diameter, 10 cm depth) per plot were taken and extracted in a high gradient Kempson extractor at the Justus‐Liebig University in Gießen, Germany. A subsample from each composite sample was taken from within a 5 × 5 cm ring and subsequently transferred into ≥ 98% pure ethanol. Identification (Appendix S1) was performed on a total of 1539 processed Collembola specimens, which could be assigned to 60 taxa: 48 to species level, 12 to genus, 312 juveniles were identified to family level and three specimens remained unidentified.

### 2.4 StrataSeq Dataset

Samples for demonstrating the StrataSeq workflow were taken in April 2021. On each of the same 150 grassland plots, four soil cores were taken with 5 × 5 cm coring rings, including litter and the first millimeters of vegetation. The microarthropod extraction was done using the MacFadyen apparatus at the Senckenberg Natural History Museum in Görlitz, Germany, over a duration of 10 days. The content was directly extracted into glass vessels filled with ≥ 98% pure ethanol. The four extracts of each plot were pooled and split into random halves. One half of each sample was retained for potential reference genome generation, while the other half was stored for a metagenomic follow‐up project.

#### 2.4.1 Habitat Stratified Sample Subsetting

A subset of the 150 samples was chosen based on three stratifying variables: region, main soil type and LUI (Blüthgen et al. 2012; Vogt et al. 2019). LUI was calculated for each of the plots with a dedicated tool for each year between 2008 and 2020 (Ostrowski et al. 2020) and then averaged for each plot. Nine main soil types were identified, with only one, cambisol, occurring in all three regions. Two main soil types occurred in the Swabian Albs (leptosol and cambisol), three in Hainich‐Dün (vertisol, stagnosol and cambisol) and six in Schorfheide‐Chorin (gleysol, type 1 histosol, type 2 drained and degraded histosol, luvisol, albeluvisol and cambisol). A high and a low LUI plot were selected by randomly choosing a plot from the lowest and highest quartiles of the land‐use intensities recorded in each region and soil type (Table S2; Merges et al. 2025). With one low and one high LUI plot per main soil type per region, the final habitat‐stratified sample subset consisted of 22 samples.

We visualised differences in community composition explained by the stratifying variables using non‐metric multidimensional scaling (NMDS) implemented in the vegan package v2.6‐6.1 (Oksanen et al. 2024) in R v4.4.3. As community data, we used specimen counts of morphospecies from the final habitat‐stratified subset (22 of the 150 samples). The statistical significance of region, main soil type and LUI was tested with permutational multivariate analysis of variance (PERMANOVA) as implemented in the adonis2 function of vegan (community~region + main soil type + LUI; distance metric: Bray–Curtis, 999 iterations). We did not control for differences in sample sizes and total specimen numbers because specimens were collected with comparable effort at each site, and observed abundance differences are therefore expected to reflect environmental gradients rather than sampling effects. We used a linear model to evaluate richness differences of morphospecies within the final habitat‐stratified sample subset (richness~region + main soil type + LUI). Data used for benchmarking, and R analysis and visualisation scripts are available from the Figshare Digital Repository https://doi.org/10.6084/m9.figshare.28622285 (Merges et al. 2025).

#### 2.4.2 Prioritising Morphospecies

The Collembola specimens of each of the 22, final, habitat‐stratified subsets were grouped into morphospecies along typical traits: adult body size, relative size and shapes of tagmata and appendages (antennae, legs, furca, ventral tube), development of eye patch, pigmentation. Notes on the number of specimens and their integrity were taken accordingly for microscopic slide preparations and later potential DNA extractions for reference genomes. The 22 habitat subset samples all contained Collembola with a total number of 2478 specimens (in contrast to the benchmarking dataset, all specimens in the sample were evaluated, without subsampling). These were grouped into 31 different morphospecies in 181 pools of morphospecies for all 22 habitat subsets (Tables S1 and S3) (Merges et al. 2025). Individual samples contained 3–13 different morphospecies (8.4 morphospecies on average). The two most common morphospecies (B: 989 and A: 225 specimens) both occurred in 15 of the 22, final, habitat‐stratified subsets, making up 50% of all specimens in the StrataSeq dataset. The five least common morphospecies occurred only in one subset each with abundances of 1 to 19 specimens (Table S1) (Merges et al. 2025). Morphospecies were selected for detailed taxonomic identification if they occurred in more than three samples. A single morphospecies occurring in a single sample was also identified because of its relatively high abundance (19 specimens). Altogether, 19 morphospecies were selected for detailed identification (Table 1 and Table S1).

**TABLE 1:** Collembola species identified with the StrataSeq workflow and the benchmarking dataset.

Order	Family	Species	StrataSeq						Benchmarking	
			Detected	Morphospecies	Count	Sequenced	Accession	Vouchers	Detected	Count
Neelipleona	Neelidae	*Megalothorax willemi*	Yes	O	9	Yes	SRX26802800	BE‐COLL‐001		
Symphypleona	Arrhopalitidae	*Arrhopalites caecus*							Yes	2
	Bourletiellidae	*Bourletiella* sp.							Yes	2
	Dicyrtomidae	*Dicyrtomina minuta*							Yes	5
	Katiannidae	*Sminthurinus aureus*	Yes	H	36	No	GCA_034698265.1		Yes	9
		*Sminthurinus elegans*	Yes	Sb	19	No	GCA_034698325.1		Yes	20
		*Sminthurinus alpinus*							Yes	2
		*Sminthurinus signatus*							Yes	7
		*Sminthurinus* sp.							Yes	18
	Sminthuridae	*Sminthurus* sp.	Yes	M	59	No	^a^		Yes	2
		*Sminthurus viridis*	Yes			No	GCA_965194885.1			
		*Sminthurus wahlgreni*							Yes	22
		*Sphaeridia pumilis*	Yes	V	194	No	GCA_034698345.1		Yes	41
Poduromorpha	Brachystomellidae	*Brachystomella parvula*	Yes	C (C + Ca)	133	No	GCA_019776545.1		Yes	8
	Hypogastruridae	*Ceratophysella denticulata*							Yes	9
		*Ceratophysella sp*							Yes	8
		*Willemia similis*							Yes	1
		*Hypogastrura* sp.							Yes	3
		*Hypogastrura vernalis*							Yes	7

	Neanuridae	*Friesea truncata*							Yes	9
		*Micranurida pygmaea*							Yes	1
		*Pseudachorutes corticicolus*							Yes	14
		*Pseudachorutes sp*							Yes	5
	Tullbergiidae	*Mesaphorura critica*	Yes	K	90	Yes	SRX26802793	BE‐COLL‐007	Yes	11
		*Mesaphorura hylophila*	Yes			Yes	SRX26802803	^b^		
		*Mesaphorura krausbaueri*	Yes			Yes	SRX26802804	BE‐COLL‐008	Yes	17
		*Mesaphorura* sp.	Yes			No	^a^	BE‐COLL‐009	Yes	1
		*Metaphorura incisa*	Yes			Yes	SRX26802798	BE‐COLL‐010		
		*Metaphorura affinis*	Yes			Yes	SRX26802797	BE‐COLL‐011	Yes	38
		*Stenaphorura quadrispina*	Yes			Yes	SRX26802796	BE‐COLL‐012	Yes	8
		*Mesaphorura macrochaeta*							Yes	13
		*Mesaphorura sylvatica*							Yes	6
*Mesaphorura yosii*								Yes	1

	Onychiuridae	*Onychiurus* sp.	Yes	A	225	Yes	SRX26802807	BE‐COLL‐013		
		*Protaphorura bicampata*	Yes			No	^c^	BE‐COLL‐014		
		*Protaphorura campata*	Yes			Yes	SRX26802808	BE‐COLL‐015		
		*Protaphorura cancellata*	Yes			Yes	SRX26802805	BE‐COLL‐016		
		*Protaphorura* sp.	Yes			No			Yes	5
		*Protaphorura subuliginata*	Yes			Yes	SRX26802792	BE‐COLL‐018		
		*Protaphorura armata*	Yes			No	GCA_034700305.1		Yes	64
		*Onychiurus cebennarius*							Yes	8
*Onychiurus edinensis*								Yes	2
Entomobryomorpha	Entomobryidae	*Lepidocyrtus cyaneus*	Yes	G	62	No	GCA_034696785.1		Yes	58
		*Lepidocyrtus lanuginosus*	Yes	D	71	Yes	SRX26802809	BE‐COLL‐021	Yes	21
		*Lepidocyrtus pallidus*	Yes			No	^c^	BE‐COLL‐022		
		*Lepidocyrtus* sp. *‐ possibly lignorum*	Yes			No	GCA_034696835.1			
		*Lepidocyrtus* sp.	Yes			No	^a^	BE‐COLL‐023	Yes	34
		*Lepidocyrtus lignorum*							Yes	12
		*Lepidocyrtus curvicollis*							Yes	1
	
		*Entomobrya multifasciata*							Yes	10
		*Entomobrya nivalis*							Yes	1
		*Entomobrya* sp.							Yes	29
		*Pseudosinella alba*	Yes	F	37	No	GCA_034700485.1		Yes	12
	*Pseudosinella immaculata*							Yes	2
	Isotomidae	*Cyphoderus albinus*	Yes	U	19	No	GCA_965194875.1			
		*Desoria duodecemoculata*							Yes	7
		*Folsomia fimetaria*	Yes	I	46	No	GCA_034695645.1		Yes	2
		*Folsomia manolachei*	Yes	L	149	Yes	SRX26802802	BE‐COLL‐027	Yes	51
		*Folsomia quadrioculata*	Yes			Yes	SRX26802810	BE‐COLL‐028	Yes	48
		*Folsomia lawrencei*							Yes	6
		*Folsomia* sp.							Yes	1
		*Folsomides parvulus*							Yes	7
		*Isotoma caerulea*	Yes	E	37	No	GCA_034696405.1			
		*Isotoma* sp. *(prob. anglicana or cearulea)*	Yes			No	GCA_034694685.1			
		*Isotoma viridis*	Yes			No	GCA_034696425.1		Yes	6
		*Isotomurus* sp.	Yes			No				
		*Isotoma anglicana*							Yes	55
*Isotomurus fucicola*								Yes	41


		*Isotomiella minor*							Yes	29
		*Isotomodes productus*	Yes	Q	21	Yes	SRX26802799	BE‐COLL‐033	Yes	42
		*Parisotoma ekmani*	Yes	Ia	40	No	^c^	BE‐COLL‐034		
		*Cryptopygus bipunctatus*	Yes			Yes	SRX26802801	BE‐COLL‐035	Yes	4
		*Cryptopygus ponticus*							Yes	1
		*Parisotoma notabilis*	Yes	B	989	Yes	SRX26802806	BE‐COLL‐036	Yes	366
*Proisotoma minima*								Yes	1
*Parisotoma* sp.							Yes	3

*Note:* Species are marked as “Sequenced” if genome data was generated using the StrataSeq workflow. Existing genome resources (GenBank accession numbers) are provided for the StrataSeq species. Remains of genome‐sequenced specimens (cleared, slide‐mounted skins were deposited as vouchers in the Apterygota collection of the Senckenberg Museum of Natural History Görlitz).

^a^ Species without existing genome data for which StrataSeq‐prioritised genome sequencing failed.

^b^ Specimens identifiable only to genus level with already available genome data for these genera, or generated after StrataSeq prioritisation.

^c^ Voucher could not be recovered after DNA extraction.

#### 2.4.3 Detailed Morphological Identification

Starting from the most common morphospecies pool with the highest number of adult and intact specimens, adult specimens were picked randomly for microscopy slide preparation (Appendix S1). For morphospecies occurring in sufficient quantity in multiple samples, further specimens were prepared from other locations, beginning with those coming from the most distinct habitats (i.e., those differing in all three stratifying variables). This was done until no new species were found in another sample, or until only single specimens of new species were detected. A total of 279 Collembola specimens of the 19 morphospecies were microscopically identified, 30 taxa to species level and nine to genus level (Table 1 and Table S4) (Merges et al. 2025). Microscopic identification revealed that 11 of the 19 morphospecies contained only a single species and 15 morphospecies contained only a single genus. The two most ambiguous morphospecies contained multiple genera and species, both belonging to euedaphic groups of Collembola (Onychiuridae and Tullbergiidae) with colourless skin, inconspicuous extremities and similar, slender shapes.

#### 2.4.4 Reference DNA Sequence Generation

The list of identified species was compared to available genomes on NCBI and to a designated soil invertebrate database (Metainvert) (Collins et al. 2023). Of the 39 identified taxa, no public genome sequences were available for 14 taxa (Table 1), including two whole genera, *Onychiurus* and *Metaphorura*. Currently, 189 genomes of Collembola are available on NCBI, representing 148 distinct species (as of 29.9.2025). A total of 66 individuals were picked for genome sequencing (Appendix S1). Genome sequences could be generated for 17 taxa. Generated genome sequence data is available at the National Center for Biotechnology Information (NCBI) as bioproject PRJNA1188452.

## 3 Results

In the following, we evaluate and benchmark StrataSeq as a conceptual workflow, focusing on its efficiency and representativeness relative to exhaustive identification, rather than presenting a complete molecular database.

### 3.1 Habitat Stratified Subsetting

**FIGURE 2:**
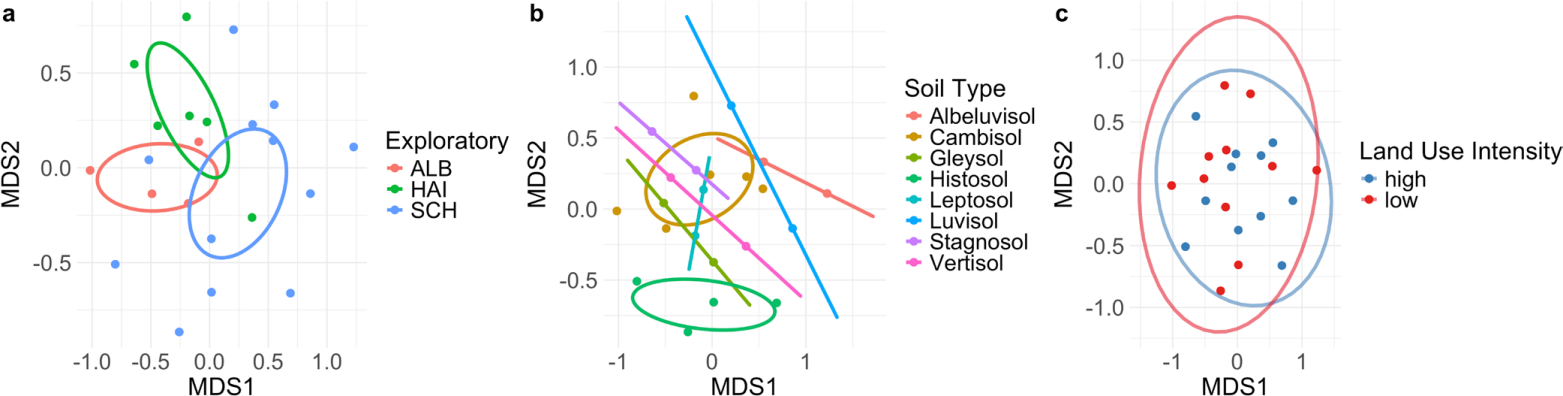
Ordination plot showing the differences in morphospecies compositions between the 22 habitat subsetting samples according to the three strata: (a) regions (Alb = Swabian Albs, Hai = Hainich‐Dün, Sch = Schorfheide‐Chorin), (b) main soil types and (c) land‐use intensity. LUI was re‐coded as high or low for visualisation purposes only (high: LUI > average, low: LUI ≤ average).

Analysis of compositional turnover of morphospecies showed that only the region, but not main soil types or land‐use intensity, were the relevant drivers of community composition (Figure 2, NMDS) implemented in vegan, R, with factor significance tested with PERMANOVA as implemented in the adonis2 function (region: *R* ^2^ = 0.17, *p* = 0.008, main soil types: *R* ^2^ = 0.336, *p* = 0.192, land‐use intensity: *R* ^2^ = 0.042, *p* = 0.413). Morphospecies richness was statistically significantly different among the regions (lowest in Schorfheide‐Chorin, comparably higher in the Swabian Albs and Hainich‐Dün; degrees of freedom df = 2, *F* = 5.91, *p* = 0.018). Soil type and land‐use intensity were not statistically significant predictors of morphospecies richness.

Most variation in community composition resulted from the first stratifying factor. Interestingly, neither soil type nor land use were important drivers of community composition in the StrataSeq samples. This is similar to previous results on the same grassland plots also found that land use only weakly affected many soil organisms (Le Provost et al. 2021). This is in contrast with other studies which report strong land‐use effects, especially in systems with tillage (Tsiafouli et al. 2015). We did not consider several potentially important factors during the stratified subsampling. For example, soil moisture and temperature likely influence turnover, but Collembola are likely adapted to rapid fluctuations of these parameters. The inclusion of further stratifying factors would have led to higher workload, while capturing increasingly similar communities. The lack of statistical significance of soil type and land‐use effects in the present results emphasises that stratification was an efficient way to capture community turnover and thus maximise variation among samples. These analyses illustrate how habitat‐stratified subsetting captures major axes of community variation, a decision step in the workflow, rather than aiming to resolve full taxonomic inventories.

### 3.2 Benchmarking

Here we benchmark StrataSeq against a traditional identification approach to evaluate efficiency, rather than to produce a final, complete molecular database. These results benchmark the StrataSeq workflow as a decision framework rather than as an automated pipeline. The workflow relies on human judgement at key steps, particularly in morphospecies sorting and prioritisation.

**FIGURE 3:**
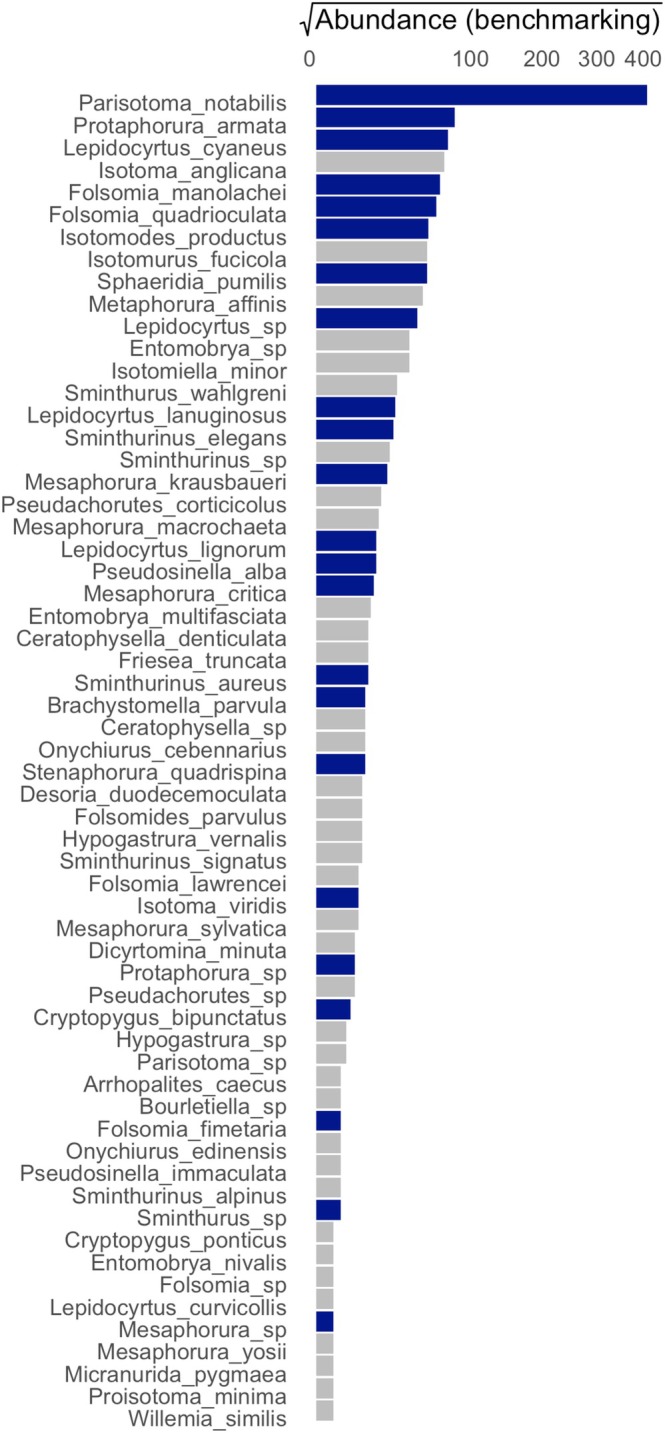
Abundance rank distribution of Collembola species in the benchmarking dataset. Species with blue bars are covered by the StrataSeq dataset, species in grey are not.

With respect to the effort spent on detailed morphological identification, a total of 1539 single individuals were prepared on microscopic slides and morphologically identified for the benchmarking dataset. Similarly, detailed identification was done only on 345 individuals for the StrataSeq dataset. This is about 22% of the effort spent on detailed morphological identification in StrataSeq, in comparison to generating the benchmarking dataset. In the benchmarking dataset, 1219 individuals could be identified to species or genus level, resulting in a taxon list of 48 species and 12 genera (Table S5—benchmarking dataset) (Merges et al. 2025). The StrataSeq workflow resulted in a taxon list of 30 species and nine genera (Table S4) (Merges et al. 2025). The overlap was 23 taxa (species or genera) between the StrataSeq and benchmarking datasets. About 62% of the *benchmarking* taxa (37) were not present in the StrataSeq dataset. About 41% of the taxa from the StrataSeq workflow (16) were not present in the benchmarking dataset. However, ecological count data is strongly skewed (O'Hara and Kotze 2010). This is also visible in the abundance densities of species in the benchmarking dataset (Figure 3): over 65% (804) of all individuals belong to the 10 most abundant species. When accounting for the skewed abundance distributions, the species list produced by StrataSeq covered over 69% of the individuals from the benchmarking dataset (Figure 3).

Accordingly, our aim here is to illustrate the efficiency of the framework, not to produce a complete molecular database. Our benchmarking clearly demonstrates that StrataSeq significantly reduces the required identification effort but captures a representative proportion (69%) of springtail biodiversity, compared to exhaustive identification. This highlights the scope of StrataSeq as a set of guiding principles for efficient prioritisation. The results should be interpreted as a proof‐of‐concept demonstration of workflow efficiency.

## 4 Discussion

We presented a conceptual workflow that relies on human judgement at key decision points, designed to guide and prioritise efforts to generate molecular reference databases within the frame of regular community ecology or biomonitoring studies. The workflow minimises workload but allows for representative assessment of biodiversity. The workflow is a novel and effective combination of four basic steps that require human expert judgement. These steps cannot be automated in a computational sense. Rather, they are key decisions that help researchers focus limited taxonomic and sequencing resources most effectively. First, the study area and sampling are stratified by habitat gradients known to differentiate the target taxa. This leads to a minimal subset of samples comprising the maximal diversity of the target taxa to be found within all study sites, and minimising the need to identify the same species repeatedly in many samples. Second, morphospecies are used as indicators for the actual species diversity to guide the subsequent morphological identification. This aids the targeted selection of single specimens for the laborious task of species identification. Third, common morphospecies are prioritised for morphological identification. Effort invested in the identification of rarer species can be weighed against available capacity. Fourth, species that lack molecular reference data are sequenced, and sequences are uploaded into public databases as permanent resources for future studies and monitoring projects.

All steps of the workflow are adaptable to different macro‐ and microorganism groups with some considerations. Habitat‐stratified subsetting solely depends on the organism group of interest and the questions that should be answered in a project. It may be adapted to both spatial and temporal settings. A precondition is the availability of environmental data (e.g., distinct vegetation types), which is frequently collected during studies. While not every environmental variable may be readily measurable, a multitude of environmental data (e.g., climate, soil type, elevation, land cover) is accessible through open sources. These may also serve as proxies for relevant environmental factors.

Prioritising morphospecies is suitable for many organism groups where external morphological characteristics allow meaningful visual grouping, such as many plants, insects and other terrestrial and aquatic arthropods (e.g., springtails, mites, spiders, or diaptomids). This prioritisation is useful to streamline and limit the identification effort, in combination with priorities set for further identification (e.g., common over rare species). If feasible, image‐based classification tools or machine learning could further assist in the sorting of morphospecies to group visually similar specimens prior to expert validation. Limiting the number of specimens allows for more time for thorough identification of each specimen, which is important for resolving the taxonomy of widespread and hard‐to‐distinguish morphospecies. For example, the StrataSeq workflow detected five species belonging to the second most common morphospecies (Table 1) (Merges et al. 2025), mainly from the *Protaphorura* genus. In contrast, only a single species (*Protaphorura armata*) was identified in the benchmarking dataset. When aiming for the generation and public provision of new molecular resources, the time gained to focus on thorough identification is of utmost importance, even if this comes at the cost of limiting the number of morphospecies that can be thoroughly investigated. StrataSeq does not replace traditional taxonomic work or deliver a database, but serves as a framework for prioritisation and efficiency. We see two ways to adapt this step to microorganisms, where morphological identification is difficult. A possible top‐down approach would be to specifically target selected taxa with fluorescence in situ hybridisation (FISH) and selected gene probes. This will allow sorting of specific, fluorescently labelled cells by flow cytometry and the generation of single cell assembled genomes (SAG). Alternatively, a possible bottom‐up approach would be to sequence the samples selected by habitat‐stratified subsetting by shotgun metagenomics, and generate metagenome assembled genomes (MAG) of the common bacterial and fungal taxa. SAGs and MAGs both inherently prioritise common microbes and combining their generation with habitat‐stratified subsetting will help to explore microbial biodiversity in a systematic and targeted manner. However, prioritising common species over completeness in species inventories comes at a price (Bennett et al. 2014; Klibansky et al. 2017), as rare species would not be sampled until the database is close to completion. Such rare species, especially microorganisms, might be very important for ecosystem functioning. For example, some microorganisms, which are rare most of the time, might become dominant for very short time periods, e.g., during pathogen outbreaks, which strongly regulate ecosystems. Considering temporal strata may be important to consider species that are dominant or rare at different points in time (Marrone et al. 2023). However, as molecular reference databases become more complete, the focus for identification and resource generation will shift from more common to more rare taxa (Meier et al. 2025). This will require conceptual adjustments to the workflow. Instead of choosing sample sites from usually targeted habitats, Palmer et al. (2002) already proposed using the uniqueness of environments to find underrepresented species.

While reference DNA sequence generation is straightforward for larger organisms, special considerations are necessary when dealing with small taxa. We already mentioned single‐cell‐assembled genomes above. Similarly, with the advancement of sequencing technologies, it becomes increasingly feasible to sequence single small metazoans collected under field conditions (Collins et al. 2023; Schneider et al. 2021). This increasingly allows us to provide reference genome data for molecular identification of morphologically hard‐to‐distinguish taxa, including juvenile specimens. A key limitation is that StrataSeq is only applicable when at least some faunistic or taxonomic information on the focal group is available in the region of interest. In areas lacking such foundations, the workflow cannot replace primary taxonomic research, but it can serve as a complementary framework for generating molecular references in parallel with species descriptions.

The comparison of the StrataSeq and benchmarking datasets shows that the workflow could identify 69% of springtail specimens with 22% identification effort. Workflow efficiency is even more impressive if we consider that (1) the specimens used for the comparison were collected in different years, (2) only a subset of benchmarking specimens was selected for identification and (3) identification was performed by different experts, an important predictor of differences among species lists (Straile et al. 2013). The high identification coverage of the StrataSeq workflow can be explained by the commonness and abundance of a small fraction of species over a large number of rarer ones (Figure 3). Such steeply declining species‐abundance relationships are the norm in ecological communities (Verberk 2011), and will be encountered when applying the workflow to other taxonomic groups and study sites.

## 5 Conclusions

Ecological studies of hard‐to‐identify species rarely have the resources to generate comprehensive DNA sequence databases that taxonomically cover all species within sampled communities in the whole region of interest. There is therefore a need for methods that optimise the efficient creation of sequence databases. The application of the StrataSeq workflow could potentially help to rapidly improve existing molecular database resources in an ecologically meaningful manner within the timeframe of regular community ecology and biomonitoring studies. As global change and pressures on ecosystems and ecological communities accelerate, improvements in the provisioning of missing molecular resources are needed to facilitate biomonitoring efforts and insight generation into ecosystem trends and stress responses. Overall, StrataSeq should be understood as a guiding framework for efficient reference database development, rather than as a stand‐alone molecular or computational method. StrataSeq is based on simply adaptable general principles that make it widely applicable for other taxa across the tree of life in both aquatic and terrestrial environments.

## Author Contributions

**Anna K. Merges:** conceptualization (equal), investigation (equal), methodology (equal), visualization (equal), writing – original draft (equal). **Peter Manning:** conceptualization (equal), funding acquisition (equal), methodology (equal), supervision (equal), writing – original draft (equal), writing – review and editing (equal). **Dennis Baulechner:** investigation (equal). **Katharina John:** investigation (equal). **Andrey Zaitsev:** investigation (equal). **Volkmar Wolters:** conceptualization (equal), methodology (equal), supervision (equal). **Damian Baranski:** investigation (equal). **Hans‐Peter Grossart:** writing – original draft (equal), writing – review and editing (equal). **Jason Woodhouse:** writing – original draft (equal), writing – review and editing (equal). **Clément Schneider:** conceptualization (equal), funding acquisition (equal), project administration (equal), supervision (equal), writing – review and editing (equal). **Miklós Bálint:** conceptualization (equal), funding acquisition (equal), methodology (equal), project administration (equal), supervision (equal), visualization (equal), writing – original draft (equal), writing – review and editing (equal).

## Conflicts of Interest

The authors declare no conflicts of interest.

## Supporting information


**Table S1:** Morphospecies and their abundances in 22 habitat subsets in the habitat subsetting dataset (HSD).

**Table S2:** Grassland sites of the Biodiversity Exploratories: location, region, soil type and land‐use intensity.

**Table S3:** Description of HSD morphospecies.

**Table S4:** Detailed species identifications of common Collembola from the habitat‐stratified samples.

**Table S5:** Species identified in the benchmarking dataset.

**Appendix S1:** ece372375‐sup‐0006‐AppendixS1.docx.

**Appendix S2:** ece372375‐sup‐0007‐AppendixS2.R.

## Data Availability

Generated genome sequence data is available at the National Center for Biotechnology Information (NCBI) as bioproject PRJNA1188452. Sequence data is embargoed until 30.6.2025 or manuscript publication. Data used for benchmarking, and R analysis and visualisation scripts are available from the Figshare Digital Repository https://doi.org/10.6084/m9.figshare.28622285 (Arana et al. 2024).
