# Supplementary material for: StrataSeq: A Workflow for Rapid Development of Molecular Databases for Hard‐To‐Identify Species
Source: Ecol Evol. 2025 Oct 23;15(10):e72375. doi: 10.1002/ece3.72375 (PMC12547479; doi:10.1002/ece3.72375)
Supplement: Supplementary file 6 — Appendix S1: ece372375‐sup‐0006‐AppendixS1.docx. [file ECE3-15-e72375-s002.docx]

# Supporting Information

### Soil type and land use intensity

For the StrataSeq workflow, data on main soil types and land-use intensities were generated by the Biodiversity Exploratories core projects [(Ostrowski et al., 2020)](https://paperpile.com/c/AO3f7o/b8bZ). The land-use intensity data was taken annually from 2008 until 2020, calculated as a mean score of standardised values of fertilisation input, grazing intensity and mowing frequency and normalised at the regional level [(Blüthgen et al., 2012)](https://paperpile.com/c/AO3f7o/3tro). In some cases, several plots had similarly high or low land-use intensity after subsetting them by main soil types per region, and some plots with high or low mean land use intensity values showed more fluctuation in land-use over the years than others with similar scores. Here, we selected samples from plots with the least fluctuation in intensity over the years within the 15% and 85% quantile of the averaged land-use intensity index values.

## Morphological identification

Specimens were cleared in lactic acid. Specimens in the benchmarking dataset were prepared on microscopic slides and identified to species level [(Bretfeld, 1999; Fjellberg, 1998, 2007b; Potapov, 2001; Thibaud et al., 2004)](https://paperpile.com/c/AO3f7o/ewi9R+D5CQ+Q2Wjl+U1IER+f5kwx). Strongly pigmented specimens were additionally cleared within the StrataSeq workflow using KOH tablets (Carl Roth, Karlsruhe, Germany) dissolved in a few drops of water. The cleared specimens were mounted on microscopic slides using Marc André II mounting medium, one specimen per slide. Identification was done using keys [(Dunger & Schlitt, 2011; Fjellberg, 1998, 2007a)](https://paperpile.com/c/AO3f7o/XtHR+D5CQ+7JVx). Due to complications in slide preparation and difficulty in identification, ambiguities for two of the common species, *Isotoma anglicana* and *Metaphorura affinis* arose in step 3 of the workflow. Therefore, these species are not listed in our dataset. However, we suspect that these species were actually among the benchmarking specimens as they are common, and respective specimens were picked from samples of several habitats. Including them into the StrataSeq dataset would increase the identification coverage to over 75% of the benchmarking specimens. As reference genomes for both species already exist, however, spending more time on identification would have been contrary to our aim of efficient databasing.

## Genome generation

DNA from specimens selected for genome sequencing was extracted using the MagAttract kit (QIAGEN, Hilden, Germany), without crushing the specimens. Lysis times were adjusted according to the specimen's body sizes (0.5 - 2.5 hours), frequently checking the state of lysis. After the lysis step, the skins were recovered under a binocular microscope using a mechanical pipette. The skins were then directly pipetted onto a microscopic slide prepared with Marc André II and microscopically checked for species verification. Library preparation was done in the TBG Robotics Facility at the Senckenberg Biodiversity and Climate Research Centre in Frankfurt, Germany, using the BEST protocol [(Carøe et al., 2018)](https://paperpile.com/c/AO3f7o/xbhZ). Illumina sequencing was done with Novogene (Cambridge, UK) with the Illumina Novaseq platform.

**References**

[Blüthgen, N., Dormann, C. F., Prati, D., Klaus, V. H., Kleinebecker, T., Hölzel, N., Alt, F., Boch, S., Gockel, S., Hemp, A., Müller, J., Nieschulze, J., Renner, S. C., Schöning, I., Schumacher, U., Socher, S. A., Wells, K., Birkhofer, K., Buscot, F., … Weisser, W. W. (2012). A quantitative index of land-use intensity in grasslands: Integrating mowing, grazing and fertilization. *Basic and Applied Ecology*, *13*(3), 207–220.](http://paperpile.com/b/AO3f7o/3tro)

[Bretfeld, G. (1999). *Synopses on Palaearctic Collembola*. Staatliches Museum für Naturkunde.](http://paperpile.com/b/AO3f7o/ewi9R)

[Carøe, C., Gopalakrishnan, S., Vinner, L., Mak, S. S. T., Sinding, M. H. S., Samaniego, J. A., Wales, N., Sicheritz-Pontén, T., & Gilbert, M. T. P. (2018). Single-tube library preparation for degraded DNA. *Methods in Ecology and Evolution*, *9*(2), 410–419.](http://paperpile.com/b/AO3f7o/xbhZ)

[Dunger, W., & Schlitt, B. (2011). Synopses on Palaearctic Collembola – TULLBERGIIDAE: with 248 figures and 7 tables. *Soil Organisms*, *83*(1), 1–168.](http://paperpile.com/b/AO3f7o/7JVx)

[Fjellberg, A. (1998). *Fauna Entomologica Scandinavica Volume 35. The Collembola of Fennoscandia and Denmark. Part I: Poduromorpha*. Brill, Leiden.](http://paperpile.com/b/AO3f7o/D5CQ)

[Fjellberg, A. (2007a). *The collembola of Fennoscandia and Denmark*. Brill Leiden, The Netherlands.](http://paperpile.com/b/AO3f7o/XtHR)

[Fjellberg, A. (2007b). *The Collembola of Fennoscandia and Denmark, Part II: Entomobryomorpha and Symphypleona*. Brill.](http://paperpile.com/b/AO3f7o/Q2Wjl)

[Ostrowski, A., Lorenzen, K., Petzold, E., & Schindler, S. (2020). *Land use intensity index (LUI) calculation tool of the Biodiversity Exploratories project for grassland survey data from three different regions in Germany since 2006, BEXIS 2 module*. Zenodo. https://doi.org/](http://paperpile.com/b/AO3f7o/b8bZ)[10.5281/ZENODO.3865579](http://dx.doi.org/10.5281/ZENODO.3865579)

[Potapov, M. (2001). Synopses on Palaearctic Collembola: Isotomidae. *Abhandlungen Und Berichte Des Naturkundemuseums Görlitz*, *73*(2), 603.](http://paperpile.com/b/AO3f7o/U1IER)

[Thibaud, J.-M., Schulz, H.-J., da Gama Assalino, M. M., & Schulz, H.-J. (2004). *Synopses on Palaearctic Collembola*. Staatliches Museum für Naturkunde.](http://paperpile.com/b/AO3f7o/f5kwx)
